# Should Empiric Anti-Fungals Be Administered Routinely for All Patients with Perforated Peptic Ulcers? A Critical Review of the Existing Literature

**DOI:** 10.3390/pathogens13070547

**Published:** 2024-06-28

**Authors:** Kai Siang Chan, Lee Yee Calista Tan, Sunder Balasubramaniam, Vishal G. Shelat

**Affiliations:** 1 Lee Kong Chian School of Medicine, Nanyang Technological University, Singapore 308232, Singapore; ctan255@e.ntu.edu.sg (L.Y.C.T.); vgshelat@gmail.com (V.G.S.); 2 Department of General Surgery, Tan Tock Seng Hospital, Singapore 308433, Singapore; sunder_balasubramaniam@ttsh.com.sg; 3 Yong Loo Lin School of Medicine, National University of Singapore, Singapore 117597, Singapore

**Keywords:** anti-fungal, culture, intra-abdominal sepsis, microbiology, peptic ulcer perforation

## Abstract

A perforated peptic ulcer (PPU) is a surgical emergency with a high mortality rate. PPUs cause secondary peritonitis due to bacterial and fungal peritoneal contamination. Surgery is the main treatment modality and patient’s comorbidites impacts perioperative morbidity and surgical outcomes. Even after surgery, resuscitation efforts should continue. While empiric antibiotics are recommended, the role of empiric anti-fungal treatment is unclear due to a lack of scientific evidence. This literature review demonstrated a paucity of studies evaluating the role of empiric anti-fungals in PPUs, and with conflicting results. Studies were heterogeneous in terms of patient demographics and underlying surgical pathology (PPUs vs. any gastrointestinal perforation), type of anti-fungal agent, timing of administration and duration of use. Other considerations include the need to differentiate between fungal colonization vs. invasive fungal infection. Despite positive fungal isolates from fluid culture, it is important for clinical judgement to identify the right group of patients for anti-fungal administration. Biochemistry investigations including new fungal biomarkers may help to guide management. Multidisciplinary discussions may help in decision making for this conundrum. Moving forward, further research may be conducted to select the right group of patients who may benefit from empiric anti-fungal use.

## 1. Introduction

Peptic ulcer disease (PUD) is a gastrointestinal disorder caused by an imbalance between the gastric mucosal protective factors and destructive factors [1]. It affects up to 3% of the population [2], and patients with significant risk factors tend to have a poorer prognosis. These factors include advanced age, smoking, use of non-steroidal anti-inflammatory drugs (NSAIDs) and *Helicobacter pylori* (*H. pylori*) infection [3].

Perforation is the most feared complication of PUD. The incidence of perforated peptic ulcer (PPU) is reported to be 2–10% of all patients with PUD with a high mortality risk of up to 20% [4]. Surgical management—most commonly omental patch repair (OPR)—is the only definitive treatment option for PPUs. Prognostication tools such as the Boey score and the Mannheim Peritonitis Index (MPI) have been created for perioperative risk stratification for 30-day mortality in PPUs [5,6]. Unlike generic risk scoring tools such as the Portsmouth-Physiological and Operative Severity Score for the enUmeration of Mortality and morbidity (P-POSSUM) and the National Emergency Laparotomy Audit (NELA) score [7,8], PPU-specific risk scoring tools such as the Boey score and MPI include time from perforation to admission as one of the covariates, suggesting the time-sensitive nature of PPUs [5,6].

**Figure 1:**
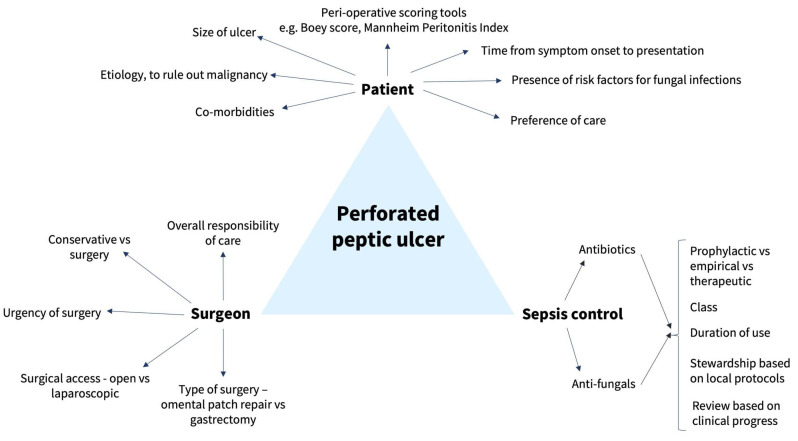
Key considerations in the management of perforated peptic ulcers.

Additionally, in PPUs, there is spillage of gastric contents into the peritoneal cavity which results in intra-peritoneal contamination with gut microbiota, consisting of bacteria and fungi, which can cause secondary peritonitis [9]. Hence, even after surgical intervention, active management including fluid resuscitation, antibiotic therapy and the monitoring of clinical progress for potential deterioration or post-operative complications is required. However, empiric anti-fungal therapy is not routinely prescribed for patients with PPUs, and we seek to understand why [10]. In view of the complexity of the management of PPUs, we have summarized the important considerations in Figure 1 for clinicians to note.

The incidence of positive fungal intra-peritoneal fluid cultures is high, ranging from 38% to 57% across various studies [11,12]. While *Candida* spp. are colonizers of the human gut and are physiological, they have the propensity to cause pathological infections and serious and life-threatening peritonitis and candidemia. Mortality for invasive candidiasis (defined as the presence of candidemia, or deep-seated infections including intra-abdominal abscess and peritonitis with or without candidemia) [13] is extremely high, ranging from 35–80% in intensive care units (ICUs) [14,15,16,17]. Then why is empiric anti-fungal therapy not routinely prescribed? A review by Huston et al. in 2019 summarized the existing literature on the role of empiric anti-fungals in PPUs and concluded a lack of evidence supporting its routine use [18]. However, new works have been published since then, and certain aspects are missing from their review—the inclusion of consensus statements from international organizations, a critical appraisal of literature based on their study methodologies and consideration of the use of adjuncts to guide clinical decision making. This review aims to provide an updated review on the use of empiric anti-fungals in PPUs with up-to-date consensus statements from international associations, and also raise important considerations for readers when interpreting the available evidence.

## 2. Definitions

It is important to define what “empiric” refers to. Various terminologies have been used for the use of anti-microbial therapy; these include “prophylactic”, “empiric” and “therapeutic”. Prophylactic is used in the context of primary prevention of infection, with no suspicion or evidence of infection at the point of clinical review. Empiric refers to a suspicion of infection using good clinical judgement and holistic assessment based on the clinical parameters guided by laboratory and radiological investigations, but not proven based on microbiology studies. Therapeutic refers to proven infection from microbiology studies. In the context of PPUs, isolation of fungus from the peritoneal fluid cultures does not necessarily imply infection and the term “empiric” will be used [12]. This is because perforation results in contamination of the intra-peritoneal cavity by the fungus which is a commensal within the oro-digestive track. This extraluminal spread of fungi does not necessarily equate to infection, though it has the potential for pathologic infection. However, any isolation of fungus from blood cultures implies an infection which warrants therapeutic anti-fungals.

## 3. Overview of Existing Guidelines on Perforated Peptic Ulcers

To our knowledge, there is only one set of guidelines thus far on the management of PPUs, i.e., the 2020 World Society of Emergency Surgery (WSES) guidelines. They provide a series of recommendations in the management of PPUs including non-operative, endoscopic, surgical and perioperative care [10]. While there are strong recommendations (Level 1C evidence) for broad-spectrum empiric antibiotics, the WSES guidelines do not recommend empiric anti-fungal therapy for all patients, but only for high-risk groups (e.g., advanced age, immunocompromised, multiple co-morbidities, prolonged ICU stay, etc.) (weak recommendation, Level 2C evidence). As there is a lack of available guidelines on the perioperative management of PPUs, we included guidelines on the role of anti-fungals in general intra-abdominal infections and/or perforated viscera and extrapolated the relevant information [19,20,21]. Table 1 summarizes the guidelines on the management of perforated viscera and/or intra-abdominal sepsis and the role of anti-fungals.

Based on the review of available guidelines, none of the guidelines suggest a role for routine empiric anti-fungals for patients with PPUs or other intra-abdominal infections. Both the WSES guidelines for PPUs and the Infectious Diseases Society of America (IDSA) guidelines for intra-abdominal infections suggest the use of anti-fungals only for patients with risk factors for candidiasis (e.g., immunocompromised, old age, presence of multiple co-morbidities, etc.). However, these risk factors are not clearly defined—e.g., the cut-off for “old” age (65, 70 or even 80 years) [22,23,24]. The liberal use of anti-fungals has downstream effects with a risk of anti-fungal resistance, adverse drug reactions or even a deleterious impact on the environment due to carbon footprints [25]. This is similar to the controversy regarding the use of routine antibiotics for prophylaxis in critically ill patients with severe acute pancreatitis [26,27]. One consideration is the rapid emergence of fungal resistance [28], which explains why existing guidelines recommend echinocandins as first-line empiric anti-fungals instead of azoles [10,13]. Another reason is the side effect and safety profile of anti-fungal use; though minimal, long-term administration may result in hepatotoxicity (for echinocandins and azoles) and adrenal insufficiency (for azoles) [29].

However, guidelines only serve as recommendations; the onus is on the surgeon to assess the level of evidence based on his or her clinical judgement and assessment of the patient’s risk profile and propensity to deteriorate. Referrals should be made to infectious disease specialists and pharmacists when warranted, e.g., the discussion of drug–drug interactions, the need for therapeutic drug monitoring, or advice on the choice of antibiotics or the addition of anti-fungal coverage in the event of clinical deterioration. In addition, despite the availability of clinical guidelines to manage these clinical conundrums, concerns about compliance with guidelines have been raised, for instance, in the management of acute pancreatitis, where more than half of patients with mild acute biliary pancreatitis received prophylactic antibiotics despite recommendations against their routine use [30]. Thus far, no studies have been performed to assess compliance with guidelines on empirical anti-fungal use in PPU. Nevertheless, we advocate that all clinicians must exercise their clinical acumen and manage patients based on their professional assessment, expertise and multidisciplinary discussions to ensure the appropriate use of anti-fungals if deemed necessary.

## 4. Current Literature on the Role of Anti-Fungals in PPUs

A literature search was performed on PubMed, Embase (Ovid) and Google Scholar on “anti-fungals”, “perforated peptic ulcer”, “intra-abdominal infection”, and “perforated viscus” on 1 March 2024. Relevant studies which performed a comparative study between the use of empiric anti-fungal therapy vs. no anti-fungals in PPUs, intra-abdominal sepsis and/or perforated viscera of gastrointestinal sources were summarized in Table 2 (study characteristics and patient demographics) and Table 3 (clinical outcomes). The size of PPUs was not reported in any of the included studies. Two main outcomes were assessed—short-term mortality and length of stay (LOS). All of the included studies assessed mortality except for the study by Barmparas et al. [31]. Other study outcomes assessed include LOS (*n* = 4 studies) [32,33,34,35], ventilator days (*n* = 3 studies) [32,33,34], and intra-abdominal abscess (*n* = 2 studies) [32,33]. Various factors analyzed in the included studies will subsequently be discussed.

### 4.1. Clinical Outcomes

#### 4.1.1. Mortality

For studies on PPUs [32,33,34,36], there were no studies which showed a significant difference in mortality between empiric anti-fungal use vs. no anti-fungal use. In contrast, studies on intra-abdominal sepsis in general were optimistic, where Lee et al. showed lower 30-day all-cause and fungal-related mortality with empiric anti-fungal use compared to culture-directed anti-fungal use [35], Vergidis et al. showed that early anti-fungal therapy was an independent predictor of lower 100-day mortality for gastrointestinal-related sepsis [37], and Sandven et al. showed near statistically significantly lower 90-day mortality with empiric single-dose intra-operative fluconazole compared to placebo treatment [38].

#### 4.1.2. Length of Stay

With regards to LOS, two studies on PPUs showed that anti-fungal use was instead associated with longer overall LOS and ICU LOS [32,34], while one study showed no difference in overall and ICU LOS between empiric anti-fungal therapy and no anti-fungal therapy [33]. A study by Lee at al. on intra-abdominal sepsis did not show a difference between empiric and culture-directed anti-fungal use on overall and ICU LOS [35]. Duration of ventilator use was statistically significantly longer with anti-fungal use compared to no anti-fungal use in a study by Chammas et al. [32]; however, this may not be clinically significant as the median was 0 days in both groups. Lastly, Lee et al. showed that empiric anti-fungal use was associated with faster overall clinical improvement (defined as improvement in hemodynamic status or normalization of temperature and leukocyte counts) and gut recovery (defined as conversion from total parenteral nutrition to enteral feeds) compared to culture-directed anti-fungal use [35]. We will be explaining the reasons for the difference in clinical outcomes reported across various studies. As shown in Table 2, clinical profiles and interventions were heterogenous in the included studies. Sources of heterogeneity include:

(a) The use of a variety of anti-fungal regimes (either fluconazole, echinocandin or amphotericin B)
(b) timing of anti-fungal administration—post-operative vs. intra-operative (only in the study by Sandven et al. [38]) vs. pre-operative (only in the study by Horn et al. [33])
(c) surgical pathology—PPUs only [31,32,33,34,36] vs. intra-abdominal sepsis of any cause, such as perforated viscera/anastomotic leaks [35,37,38]
(d) patient demographics—e.g., in all groups of patients or only in patients admitted to ICU; or only in patients with positive fungal isolates

### 4.2. Considerations on Anti-Fungal Use

#### 4.2.1. Class of Anti-Fungal Use

There are four main classes of anti-fungals available—azoles (e.g., fluconazole, voriconazole), echinocandins (e.g., anidulafungin, caspofungin), polyenes (e.g., amphotericin B, nystatin) and allylamines (e.g., terbinafine). Existing guidelines on anti-fungal use for PPU/intra-abdominal sepsis do not provide suggestions on the choice of anti-fungal for empiric use, since empiric use is not even recommended for every patient in the first place [10,13,19]. However, should empiric anti-fungal therapy be used (for high-risk patient groups), echinocandins (caspofungin/anidulafungin/micafungin) are preferred as first-line therapy for critically ill patients due to the fungicidal properties (compared to fluconazole, which is fungistatic) and lower resistance. By definition, fungicidal drugs aim to kill all fungi and reduce fungal count by 100% [39]. Realistically, fungicidal treatment is defined as an absolute reduction in fungal count by ≥99.9% due to limitations in in-vitro testing. In contrary, azoles are fungistatic, i.e., inhibit growth rather than kill pathogens; this is done via the inhibition of ergosterol synthesis—an essential component in the fungal cell wall membrane—through the inhibition of cytochrome P450-dependent 14α-lanosterol demethylase [40]. This leads to the “trailing phenomenon”, where there is persistent but reduced growth at concentrations above the minimum inhibitory concentration [41]. Hence, the guidelines suggest fluconazole only for patients who are hemodynamically stable, with azole-susceptible *Candida* infections and no prior use of azoles (in view of the risk of resistance).

Of the studies included in Table 2, there was no standardization in the class of empiric anti-fungal use, except for the study by Sandven et al. [38], which used fluconazole only (Table 2). Additionally, fluconazole was the most commonly used anti-fungal reported in each study. This heavily limits interpretation of the results due to the difference in the pharmacological properties of various classes of anti-fungals. As described above, a lack of statistical significance in mortality may be due to the lack of clinical efficacy of a choice of anti-fungals (i.e., fluconazole instead of echinocandins). This hypothesis is similarly applicable to other outcomes assessed; ICU LOS was reported to be longer with empiric anti-fungal therapy in the study by Chammas et al. and Li et al. [32,34]. This may be because the liberal use of fluconazole as an empiric anti-fungal agent was ineffective due to the incidence of azole-resistant strains. In contrast, retrospective studies are prone to selection bias, where patients who were more critically ill (who will have longer ICU LOS) were given empiric anti-fungals. Hence, these studies are of low quality, which calls for the need for better designed prospective studies to address confounding factors.

#### 4.2.2. Timing of Empiric Anti-Fungal Administration

Timing of anti-fungal administration is a consideration, but has not been widely reported in the literature, unlike antibiotics use. Early administration of antibiotics within the “golden hour” is one of the key tenets in the management of sepsis with a reduction in mortality [42]. In PPUs, empiric anti-fungals are usually given in the post-operative period if required. Instead, a study by Horn et al. compared the use of pre-operative anti-fungal use vs. no anti-fungal use but showed no difference in in-hospital mortality, overall LOS, ICU LOS, 30-day readmission, incidence of intra-abdominal abscess and fungemia [33]. They also reported a similar incidence of post-operative anti-fungal use in patients who received pre-operative anti-fungal therapy compared to those who did not (75% vs. 77%, *p* = 1.0).

However, there are studies which support the use of early empiric anti-fungals. An RCT by Sandven et al. on patients with perforated viscera showed that a single dose of intra-operative fluconazole showed near statistically significantly lower 90-day mortality compared to no fluconazole use (OR 0.21, 95% CI: 0.04–1.06, *p* = 0.059) [38]. Similarly, Vergidis et al. showed that early empiric anti-fungal therapy (defined as the administration of anti-fungals within 5 days of positive peritoneal fungal fluid culture) was independently associated with lower 100-day mortality (OR 0.36, 95% CI: 0.13–0.96, *p* = 0.042) for intra-abdominal sepsis secondary to gastrointestinal sources [37].

The difference in findings above may be explained by the difference in study population rather than timing of empiric anti-fungal administration—Horn et al. studied PPUs only [33], while Sandven et al. and Vergidis et al. studied all patients with perforated viscera [37,38]. Positive peritoneal fluid cultures for patients with perforated viscera and intra-abdominal sepsis has been shown to be associated with a prolonged ICU stay of ≥10 days; Sandven et al. showed that the presence of intra-operative positive yeast cultures was associated with a prolonged ICU stay of ≥10 days (OR 5.4, 95% CI: 1.5–19.3) for intra-abdominal sepsis [38]. Similarly, Jindal et al. showed that positive fungal fluid culture was associated with increased surgical site infection, an ICU stay of >5 days, and mortality in patients with perforated viscera [43]. In contrary, in a cohort of patients with PPUs, multivariate analysis by Kwan et al. showed that positive peritoneal fungal isolate was not associated with worse clinical outcomes [12]. It is possible that studies which showed better results with empiric anti-fungals may be due to the inclusion of patients with perforated viscera from any gastrointestinal source and not PPUs alone. We cannot conclude, based on the above evidence, that early pre-operative empiric anti-fungal therapy does not improve post-operative outcomes in PPUs.

#### 4.2.3. Duration of Empiric Anti-Fungal Administration

The duration of empiric anti-fungal use in the reviewed studies was also heterogenous, ranging from a single intra-operative dose to a mean of 14 days (Table 2). The WSES 2020 guidelines for PPUs recommend a 7–10 day course of fungistatic anti-fungals for critically ill patients at low risk of invasive candidiasis if empiric anti-fungals are warranted [10], whereas the duration depends on the extent of organ involvement for high-risk patient groups. Additionally, patients with candidemia should have at least 14 days of anti-fungals after documented evidence of the clearance of candidemia. On the other hand, the IDSA 2016 guidelines recommend that anti-fungals be stopped after 4–5 days if there is no observed clinical response with no evidence of invasive candidiasis (strong recommendation, low-quality evidence) [13]. However, it is possible that the lack of benefit observed from empiric anti-fungals is due to inadequate duration of use. For instance, the mean duration of empiric anti-fungal use in the study by Lee et al. was 14 days (vs. 15 days for culture-directed use) [35]; they showed lower 30-day all-cause and fungal-related mortality with empiric anti-fungal use compared to culture-directed use. However, as described above, a possible confounding factor is the inclusion of all patients with intra-abdominal sepsis. The sample size was also small (33 patients with empiric anti-fungal use, 15 patients with culture-directed use).

In view of the lack of evidence, we feel that it is the ethical responsibility of the clinician to determine when empiric anti-fungal therapy is deemed futile and make a decision based on good clinical judgement to stop in order to avoid harm to patients—e.g., adverse drug reactions, or the development of anti-fungal resistance. This decision may be made after consulting with critical care or infectious disease physicians.

### 4.3. Type of Studies Included

On top of the above study considerations, it is also important to look at the nature of the study. Retrospective studies are at risk of selection bias. Of the studies included, there was one randomized controlled trial, one prospective study, and six retrospective studies, of which only one study had performed propensity score matching (PSM). Given the emergent nature of PPUs which warrants immediate surgical treatment, RCTs are challenging to conduct—this has been shown by a recent meta-analysis on comparing laparoscopy versus open OPR in PPUs, with only four of 29 studies included which were RCTs [44]. In these circumstances, an alternative (though inferior) option would be to conduct propensity score matching (PSM) to create a similar cohort of patients with comparable demographics to reduce confounders [45]. Both Chammas et al. and Li et al. used PSM to create two cohorts with comparable demographics to reduce the effect of confounders [32,34]. Their studies showed no difference in mortality between empiric vs. no empiric anti-fungal use; they also showed worse outcomes with empiric anti-fungals, with longer overall LOS (in the cohort by Chammas et al. [32]) and more patients with prolonged ICU LOS (in the cohort by Li et al. [34]). The sample size, however, is small (a total of 154 in the PSM cohort for both studies). Future studies should be prospectively designed, or if not, should perform PSM to improve the quality of the studies.

## 5. Important Considerations to Direct Empiric Anti-Fungal Use

### 5.1. Biochemistry and Microbiology Results

Determining the presence of fungal infection is largely driven by culture results and the clinical progress of patients. In our institution, preliminary results take about 2–3 days. This presents a window of opportunity for early empiric anti-fungal use. However, surgeons should also review biochemistry results to decide (i) whether to start empiric anti-fungals before the culture results are out, (ii) if empiric anti-fungals are started, when to stop, and (iii) whether to start anti-fungals for all patients with positive fungal fluid cultures.

Biochemistry results are readily available within a few hours from the initial blood taking and serve as good adjuncts to guide clinical management. Common inflammatory markers include white blood cell count (WCC), C-reactive protein (CRP) and procalcitonin. Both leukocytosis and leukopenia are possible in fungal infections due to systemic inflammation. Unlike bacterial infections where there is a predominance of neutrophils, eosinophilia and monocytosis are also observed in fungal infections [46]. CRP and procalcitonin are adjuncts used in the diagnosis of infections. For instance, leukocytosis may be observed in patients on steroidal treatment, but CRP is unlikely to be raised in the absence of an infection. Procalcitonin is a biomarker increasingly used to differentiate between bacterial and non-bacterial courses of inflammation and/or infection, with higher sensitivity and specificity compared to CRP [47].

The combined utility of high CRP and low procalcitonin has been shown to accurately diagnose systemic fungal infections even in immunocompromised patients who may not be able to mount an inflammatory response, with a sensitivity and specificity of 81% and 85%, respectively [48]. Serial measurement of procalcitonin has been shown to safely guide antibiotics use in patients with acute pancreatitis with a reduction in antibiotics use without increasing the incidence of clinical infections [49]. Serial trending of inflammatory markers may similarly be performed to decide on a clinical response; for instance, persistently low procalcitonin with downtrending CRP after the initiation of empiric anti-fungals may suggest a response. However, downtrending CRP may also be due to improving bacterial infection due to source control (surgery) for PPUs and the administration of empiric antibiotics.

This difficulty in the diagnosis of fungal infection and identifying whether there is a clinical response has led to the development of other biomarkers which may serve as useful adjuncts, including serology-based, molecular-based, and biosensor-based diagnostic tests [50]. Commercially available techniques have been described in detail by Fang et al. in the diagnosis of invasive fungal infections [50]. Two commercially available techniques—(1,3)-β-D-glucan and T2Candida^®^ (T2Biosystems, Inc., Wilmington, MA, USA)—have been used to diagnose intra-abdominal candidiasis. (1,3)-β-D-glucan is a serology test for fungal cell wall components; it has been reported to have a sensitivity of 65% and specificity of 75% for deep-seated candidiasis [51,52]. As fungi are colonizers of the gastrointestinal tract, it is possible to return a positive test without any ongoing fungal infection. Quantitative interpretation would be useful in these cases, where higher values suggest a higher possibility of a fungal infection; Talento et al. showed that proven and probable invasive fungal disease had higher mean (1,3)-β-D-glucan levels compared to those with fungal colonization [53]. Nevertheless, there are reports of high false positives in view of its cross-reactivity, which includes concurrent bacteremia. Hence, its utility in PPUs and/or intra-abdominal sepsis may be limited, since isolated bacteremia may also lead to a false positive result [54].

What is noteworthy is T2Candida^®^, a magnetic resonance and molecular-based diagnostic panel that allows for the diagnosis of candidemia through the identification of the five most common *Candida* spp. from whole blood samples in five hours [55]. It is quick to perform (<5 h) compared to traditional culture-based techniques, and has a sensitivity and specificity of 91.1% and 99.4% respectively [56]. Pilot studies on the use of T2Candida^®^ showed the successful diagnosis of invasive candidiasis in seven patients with negative blood cultures but who were proven to have fungal infections using tissue biopsy or cultures obtained from a normally sterile site. T2Candida^®^ was also reported to have diagnosed intra-abdominal candidiasis 7 days prior to the discovery of an intra-abdominal source of infection (via laparotomy and tissue biopsy) in a patient who had 12 sets of negative blood cultures [57]. This is highly relevant for PPUs, as patients may have negative fungal fluid cultures, or may have positive fungal fluid cultures due to yeast colonization but no invasive fungal infection. T2Candida^®^ may help to guide decisions on whether empiric anti-fungals should be administered. Bilir et al. developed a decision-tree model to perform a cost-benefit analysis on the use of T2Candida^®^ as an adjunct to blood culture when *Candida* infection is considered a possible diagnosis [58]. Through the model, they demonstrated a 47.6% reduction in candidemia diagnosis and treatment budget, and 60.6% candidemia-related mortality per hospital. The reduction in costs was attributed to shorter LOS and lower candida-related mortality.

### 5.2. Routine Intra-Peritoneal Fluid Cultures

The WSES 2020 guidelines recommend the collection of peritoneal cultures for both bacteria and fungi in all patients undergoing surgery (Grade 1C recommendation) [10]. Several studies have shown that the presence of fungal isolates is associated with worse clinical outcomes, such as longer LOS and increased mortality [59,60]. However, the evidence is conflicting. Univariate analysis by Kwan et al. showed that peritoneal fungal isolates was associated with ulcer size ≥ 10 mm, ICU admission, intra-abdominal collection and LOS ≥ 9 days [12]. However, multivariate analysis did not show any significant correlations.

The incidence of positive peritoneal fluid cultures and blood cultures for candidemia is low, ranging from 25–50% and 50–70% respectively [12,33,36,59,61]. Even if fluid cultures are positive, starting anti-fungal treatment may not be warranted. Should peritoneal fungal cultures be routinely sent in PPUs in view of the above evidence? Our institution similarly follows the recommendations by WSES, with a liberal policy of sending intra-operative peritoneal fluid cultures for bacteria and fungi, as this may help in subsequent decision making. However, surgeons need to be cognizant that positive fluid culture does not equate to anti-fungal treatment. Sandven et al. showed that yeast colonization was high in surgical patients with intra-abdominal perforations [62]. Out of the 40 patients with yeast present in abdominal fluid cultures, 40% had an uncomplicated post-operative course. The unnecessary administration of anti-fungals may lead to unnecessary treatment and adverse reactions from anti-fungals. Surgeons should not decide immediately based on a single positive fungal fluid culture, but instead should make a wise decision based on the patient’s clinical progress and biochemistry markers. For instance, positive fungal fluid cultures may be useful in situations when the patient is not improving clinically post-operatively and there is a plan to start anti-fungal treatment. Identification of the fungus and anti-fungal sensitivity will then help to guide the choice and duration of anti-fungal treatment.

### 5.3. Presence of Concomitant Bacterial Peritonitis

While bacterial and fungal infections are separate entities, they are not mutually exclusive. Patients with fungal peritonitis may have concomitant bacterial peritonitis due to intra-abdominal contamination. The WSES 2020 guidelines recommend the administration of broad-spectrum antibiotics for PPUs (strong recommendation, level 1C evidence) [10]. This should be broad enough to cover for Gram-positive, Gram-negative and anaerobic bacteria. Even in patients who received non-operative treatment, management with empiric antibiotics, repeated clinical assessment and nil-by-mouth with fluid replacement was reported to have been successful in 72% of 40 patients without the need for surgery [63]. Empiric broad-spectrum antibiotics are crucial in controlling sepsis secondary to PPUs. However, it has been postulated that the use of empiric antibiotics disrupts the gut microbiome and leads to fungal overgrowth [64]. The use of empiric antibiotics for suspected bacterial peritonitis may result in fungal peritonitis as well.

With insults from both bacterial and fungal peritonitis, it is natural to assume that clinical outcomes will be worse compared to isolated fungal/bacterial peritonitis. In a cohort of patients with peritonitis secondary to gastrointestinal perforation, Prakash et al. showed that patients with mixed bacterial and fungal positive cultures had higher mortality compared to those with isolated bacterial positive cultures (76.9% vs. 17.2%, *p* < 0.001) [60]. However, Li et al., who studied 133 patients with positive *Candida* spp. isolates, showed similar 30-day mortality in patients with concomitant bacterial peritonitis vs. those who did not (15.5% vs. 9.3%, *p* = 0.277) [34]. One consideration for the lack of statistical significance may be the small sample size; however, the study did not include a subgroup analysis and report on patient demographics, whether empiric anti-fungals were administered, and the choice of empiric antibiotics used for PPUs [34]. The decision to start therapeutic anti-fungals if both bacterial and fungal cultures are positive should still be based on clinical judgement in view of the lack of strong evidence.

## 6. Future Research

To summarize what has been discussed above, the literature is scarce, and routine empiric anti-fungal administration for PPUs is not recommended. Patients with risk factors for invasive fungal infection should, however, receive some form of empiric anti-fungal therapy. Further studies should therefore work along the lines of identifying the right group of patients who may benefit from empiric anti-fungal use. Current risk scoring tools like the Boey score and MPI include predictors of mortality, such as poor patient demographics and the duration and degree of peritonitis [1]. The Peptic Ulcer Perforation (PULP) score includes the use of steroids [65], which may be more relevant in predicting the need for anti-fungal treatment due to immunosuppression. However, validation studies are limited on the use of the PULP score [1].

Nucci et al. proposed an individualized approach to assessing the risk of invasive fungal disease in patients with acute leukemia which may be adopted for use in PPUs [66]. This can be broadly classified into host-related risk assessment (frailty and geriatric assessment), invasive fungal disease risk assessment (age > 65 years, high co-morbidity score, presence of organ dysfunction, severe neutropenia with absolute neutrophil count of <100/μL) and disease-related risk assessment (which can consider PPU-specific risk scoring tools such as the Boey score) [66]. Future studies should compare clinical outcomes in patients with these risk factors vs. those without, and in patients with positive fungal fluid isolates and those without. This may guide patient selection for empiric anti-fungal use.

## 7. Conclusions

PPU is a surgical emergency with high mortality. However, the post-operative course may be complicated in view of sepsis due to peritoneal contamination with bacteria and fungi. Intra-operatively, peritoneal cultures should be obtained for both bacteria and fungi. Resuscitation with fluid management and the early administration of empiric antibiotics are important as part of sepsis management. Evidence on the use of empiric anti-fungals is weak and routine empiric anti-fungals should not be administered in all patients, even for patients with positive fungal isolates as these may be colonizers. The decision to start empiric anti-fungal therapy should be based on patient factors (e.g., old age, immunocompromised), disease factors (e.g., worsening inflammatory markers despite surgical treatment and antibiotics), and clinical progress (e.g., worsening hemodynamic instability). Moving forward, further research may be conducted to select the right group of patients who may benefit from empiric anti-fungal use.

## Figures and Tables

**Table 1:** Summary of existing guidelines on the role of anti-fungals in perforated viscera and intra-abdominal infections.

Year	Professional Organization/Association	Target Group	Recommendation	Grade of Recommendation	Level of Evidence
2020	World Society of Emergency Surgery (WSES) [10]	Perforated peptic ulcers	Administration of anti-fungals as standard empiric therapy in PPUs not recommended Anti-fungals suggested for high-risk patients (e.g., immunocompromised, advanced age, co-morbidities, prolonged ICU stay, unresolved intra-abdominal infections)	Weak	Low quality
2017	World Society of Emergency Surgery (WSES) [19]	Intra-abdominal infections	Anti-fungals recommended for patients with hospital-acquired intra-abdominal infections, especially those with recent abdominal surgery or anastomotic leaks Choice of anti-fungals o Hemodynamically stable, no prior exposure to azoles: fluconazole o Critically ill or previous exposure to azoles: echinocandin	Strong	Low/very low quality
2016	Surgical Infection Society; Infectious Diseases Society of America (IDSA) [13]	Intra-abdominal infections	Anti-fungals recommended for clinical evidence of intra-abdominal infection, with significant risk factors for candidiasis	Strong	Moderate quality
			Choice of anti-fungals o Echinocandin o Fluconazole for patients with no recent azole exposure and not colonized with azole-resistant *Candida* species	Strong	Moderate quality
			o Amphotericin B if intolerant of other anti-fungals	Strong	Low quality
			Duration of use o 2 weeks for suspected invasive candidiasis in patients who improve	Weak	Low quality
			o To stop anti-fungal treatment after 4–5 days if no clinical response to anti-fungals and no subsequent evidence of invasive candidiasis	Strong	Low quality
2017	World Society of Emergency Surgery (WSES) [21]	Open abdomen	No recommendations on anti-fungals	NR	NR

ICU: Intensive care unit; NR: Not reported; PPU: Perforated peptic ulcers.

**Table 2:** Patient demographics and clinical profile of included studies evaluating the role of empiric anti-fungals in perforated peptic ulcers.

No	First Author, Year	Type of Study	Study Group	Regime of Anti-Fungal Use (Class/Duration)	Duration	No. of Patients	Type of Surgery	Fungal Culture Results
1	Boyapati, 2024 [36]	Retrospective	PPUs with surgical management	NR	NR	359 (*n* = 134 anti-fungals)	NR	*n* = 122/256 positive fungal culture (*n* = 90 with anti-fungal treatment, *n* = 32 without anti-fungal treatment)
2	Chammas, 2022 [32]	Retrospective, propensity score matched	Post-operative anti-fungal PPUs Admitted in ICU	Fluconazole/caspofungin/micafungin/amphotericin B	Median duration 7 days (IQR 4–12)	89 (*n* = 52 anti-fungals) PSM cohort: 74 (*n* = 37 in each arm)	50 (56%) underwent surgery; NR on type of surgery	NR
3	Barmparas, 2021 [31]	Retrospective	Post-operative anti-fungal PPUs with surgical management	Fluconazole/micafungin/others	Early anti-fungal: median 5 days (IQR 4)	554 (*n* = 239 early anti-fungal (≤24 h from surgery), *n* = 72 with delayed anti-fungal (>24 h from surgery)	Omental patch repair 78.3%, gastrectomy 4.5%, others 17.1%	*n* = 125/177 positive fungal culture *Candida* spp. (*n* = 76/177) Only *Candida* spp. without other organisms (*n* = 58/177)
4	Horn, 2018 [33]	Retrospective	Pre-operative anti-fungals in PPUs	85% fluconazole, 11% micafungin, 4% anidulafungin Duration NR	NR	107 (*n* = 27 anti-fungals)	all received surgery	*n* = 17/33 positive fungal culture Positive fungal culture + received pre-operative anti-fungal: 4/27 (14.8%) Positive fungal culture + no anti-fungal: 13/80 (16.3%)
5	Li, 2017 [34]	Retrospective, propensity score matched	Post-operative anti-fungal PPUs with positive *Candida* spp. isolate from peritoneal fluid	Fluconazole/echinocandin	≥3 days	133 (*n* = 57 anti-fungal) PSM cohort: 80 (*n* = 40 in each arm)	NR	All had positive fungal cultures for *Candida* spp.
6	Vergidis, 2016 [37]	Retrospective	Intra-abdominal candidiasis	Single agent o Fluconazole (*n* = 82 (50%)) o Caspofungin (*n* = 11 (7%)) o Voriconazole (1 (0.6%) Multiple agents (*n* = 31 (19%)) Sequential different agents (*n* = 29 (17.8%)	Median 14 days (range 1–88 days)	163 (*n* = 117 with early antifungals, i.e., within 5 days of positive fluid culture) 53 with secondary peritonitis (*n* = 10 from gastric/duodenum)	NR	All had positive fungal cultures for *Candida* spp.
7	Lee, 2014 [35]	Prospective	Intra-abdominal sepsis with abdominal surgery for gastrointestinal perforation, malignancy, pancreatitis, intestinal obstruction, severe peritonitis, or liver transplant Admitted to ICU Empiric vs. culture-directed anti-fungals	Empiric o Fluconazole (*n* = 18 (60%)) o Caspofungin (*n* = 9 (30%)) o Anidulafungin (*n* = 3 (10%)) o Amphotericin (*n* = 0) Culture-directed o Fluconazole (*n* = 14 (78%)) o Caspofungin (*n* = 3 (17%)) o Anidulafungin (*n* = 0) o Amphotericin (*n* = 1 (6%))	Empiric: mean 14 ± 9 days Culture-directed: mean 15 ± 7 days *p* = 0.639	48 (*n* = 33 with empiric anti-fungal, 15 with culture-directed anti-fungal)	All received; type of surgery NR	Positive cultures Empiric: 16 (53) Culture-directed: 18 (100)
8	Sandven, 2002 [38]	RCT	Perforated viscus/anastomotic leak Underwent emergency surgery Intra-operative single-dose fluconazole vs. placebo	Single-dose intra-operative fluconazole vs. placebo	Single-dose	109 (*n* = 53 with anti-fungal)	Type of surgery NR	Overall *n* = 33/109 (30.3) with positive yeast culture NR on yeast positive culture in intra-operative fluconazole vs. placebo group

All categorical variables are expressed as *n* (%); CI: Confidence interval; ICU: Intensive care unit; IQR: Interquartile range; NR: Not reported; OR: Odds ratio; PSM: Propensity score matched; RCT: Randomized controlled trial.

**Table 3:** Clinical outcomes of patients who received empiric anti-fungals in perforated peptic ulcers.

No	First Author, Year	ICU Admission	Mortality	Length of Stay	Other Outcomes
1	Boyapati, 2024 [36]	Anti-fungal use associated with ICU admission (*p* < 0.05)	Anti-fungal use not associated with inpatient mortality (*p* = 0.159)	NR	Intra-abdominal sepsis/bleeding Associated with anti-fungal use (***p* = 0.0009**) Re-operation Associated with anti-fungal use (***p* = 0.0002**) Number of complications Associated with anti-fungal use (***p* = 0.0004**)
2	Chammas, 2022 * [32]	all	In-hospital Anti-fungal use: 7 (19%) No anti-fungal use: 10 (27%) *p* = 0.58	Overall Anti-fungal use: 16 days (10–24) No anti-fungal use: median 9 days (IQR 6–13) ***p* = 0.001** ICU Anti-fungal use: 6 (2–10) days No anti-fungal use: 3 (1–5) days ***p* = 0.03**	Intra-abdominal abscess Anti-fungal use: 4 (11) No anti-fungal use: 4 (11) *p* = 1.000 Ventilator days Anti-fungal use: 0 (0–1) No anti-fungal use: 0 (0–0) ***p* = 0.001**
3	Barmparas, 2021 [31]	NR	NR	NR	Organ space infection # Early anti-fungal use: 14.2% No anti-fungal use: 13.7% Adjusted OR (for presence of pneumoperitoneum on imaging): 1.04 (95% CI: 0.64–1.70)
4	Horn, 2018 [33]	NR	In-hospital Anti-fungal use: 1 (3.7) No anti-fungal use: 4 (5.0) *p* = NS	Overall Anti-fungal use: 15.2 ± 15.4 No anti-fungal use: 13.9 ± 12.5 *p* = NS ICU Anti-fungal use: 6.7 ± 13.9 No anti-fungal use: 6.7 ± 11.1 *p* = NS	30-day readmission Anti-fungal use: 4 (14.8) No anti-fungal use: 15 (18.8) *p* = NS Intra-abdominal abscess Anti-fungal use: 2 (7.4) No anti-fungal use: 5 (6.3) *p* = NS Ventilator days Anti-fungal use: 1.4 ± 3.5 No anti-fungal use: 2.8 ± 7.0 *p* = NS
5	Li, 2017 * [34]	all	30-day Anti-fungal use: 4 (10) No anti-fungal use:6 (15) *p* = 0.45	Prolonged ICU LOS >14 days Anti-fungal use: 11 (27.5) No anti-fungal use: 4 (10.0) ***p* = 0.05**	Candidemia *n* = 0 Re-operation or abscess/leak within 14 days Anti-fungal use: 5 (12.5) No anti-fungal use: 1 (2.5) *p* = 0.20 *Prolonged ventilator use >14 days* Anti-fungal use: 8 (20.0) No anti-fungal use: 3 (7.5) *p* = 0.11
6	Vergidis, 2016 [37]	NR	100-day (all patients) Early antifungal use was not a predictor for 100-day mortality (OR: 0.74, 95% 0.36–1.56, *p* = 0.44) 100-day (gastrointestinal tract source) Early antifungal use was an independent predictor of reduced 100-day mortality (OR 0.36, 95% CI: 0.13–0.96, ***p* = 0.042)**	NR	NR
7	Lee, 2014 [35]	all	30-day all-cause Empiric: 6 (20) Culture-directed: 9 (50) OR 0.25, 95% CI: 0.069–0.905 ***p* = 0.03** 30-day fungal-related Empiric: 2 (7) Culture-directed: 6 (33) OR 0.14, 95% CI: 0.025–0.812 ***p* = 0.040**	Overall Empiric: 41.0 (5–161) Culture-directed: 47.5 (7–125) *p* = 0.906 ICU Empiric: 9 (0–53) Culture-directed: 10 (0–51) *p* = 0.925	Days to overall clinical improvement Empiric: 13 (4–48) Culture-directed: 35 (18–75) ***p* = 0.007** Days to gut recovery Empiric: 9 (0–48) Culture-directed: 18 (10–49) ***p* = 0.017**
8	Sandven, 2002 [38]	NR	90-day Empiric (intra-operative): 4 (7.5) Placebo: 8 (14.3) OR 0.21, 95% CI: 0.04–1.06 *p* = 0.059	NR	Presence of intra-operative positive yeast cultures associated with Mechanical ventilation ≥5 days: OR 7.7, 95% CI: 1.7–33.7, ***p* = 0.007** ICU stay ≥10 days: OR 5.4, 95% CI: 1.5–19.3, ***p* = 0.009** Central venous catheter ≥10 days: OR 4.0 95% CI: 1.3–12.7, ***p* = 0.018**

All continuous variables are expressed as median (interquartile range), or mean ± standard deviation unless otherwise specified; All categorical variables are expressed as *n* (%); Values in bold indicate statistical significance; * The outcomes reported in this study belong to the propensity score matched cohort; CI: Confidence interval; ICU: Intensive care unit; IQR: Interquartile range; LOS: Length of stay; NR: Not reported; NS: Not significant; OR: Odds ratio. # Defined as an event involving any part of the abdomen deeper than the fascial/muscle layers with ≥1 of the following: (A) purulent drainage from intra-abdominal drain; (B) organism(s) fluid or tissue analysis performed for the purposes of clinical diagnosis or treatment which identified the presence of organisms; (C) abscess or other evidence of infection involving the abdomen that was detected on gross anatomic or histopathological examination, or imaging suggestive of infection.
